# Association of loss of epithelial syndecan-1 with stage and local metastasis of colorectal adenocarcinomas: An immunohistochemical study of clinically annotated tumors

**DOI:** 10.1186/1471-2407-8-185

**Published:** 2008-06-30

**Authors:** Yosuke Hashimoto, Marek Skacel, Josephine C Adams

**Affiliations:** 1 Dept. of Cell Biology, Lerner Research Institute, Cleveland Clinic, Cleveland, OH, 44195, USA; 2 Dept. of Anatomic Pathology, Cleveland Clinic, Cleveland, OH, 44195, USA; 3 Dept. of Molecular Medicine, Cleveland Clinic Lerner College of Medicine, Cleveland Clinic, Cleveland, OH, 44195, USA; 4 Current Address: Shizuoka Cancer Center, Nagaizumi-cho, Sunto-gun 411-8777, Shizuoka, Japan; 5 Current Address: Molecular Pathology Laboratory, Dahl-Chase Pathology Associates, 417 State Street (suite 540), Bangor, ME, 04401, USA

## Abstract

**Background:**

Syndecan-1 is a transmembrane proteoglycan with important roles in cell-cell and cell-extracellular matrix adhesion and as a growth factor co-receptor. Syndecan-1 is highly expressed by normal epithelial cells and loss of expression has been associated with epithelial-mesenchymal transition and the transformed phenotype. Loss of epithelial syndecan-1 has been reported in human colorectal adenocarcinomas, but whether this has prognostic significance remains undecided. Here we have examined syndecan-1 expression and its potential prognostic value with reference to a clinically annotated tissue microarray for human colon adenocarcinomas.

**Methods:**

Syndecan-1 expression was examined by immunohistochemistry of a tissue microarray containing cores from 158 colorectal adenocarcinomas and 15 adenomas linked to a Cleveland Clinic, IRB-approved database with a mean clinical follow-up of 38 months. The Kaplan-Meier method was used to analyze the relationship between syndecan-1 expression and patient survival. Potential correlations between syndecan-1 expression and the candidate prognostic biomarker fascin were examined.

**Results:**

Syndecan-1 is expressed at the basolateral borders of normal colonic epithelial cells. On adenocarcinoma cells, syndecan-1 was present around cell membranes and in cytoplasm. In 87% of adenocarcinomas, syndecan-1 was decreased or absent; only 13% of patients had stained for syndecan-1 on more than 75% of tumor cells. Decreased syndecan-1 correlated with a higher TNM stage and lymph node metastasis and was more common in males (p = 0.042), but was not associated with age, tumor location or Ki67 index. Reduced tumor syndecan-1 staining also correlated with upregulation of stromal fascin (p = 0.016). Stromal syndecan-1 was observed in 16.6% of tumors. There was no difference in survival between patients with low or high levels of either tumor or stromal syndecan-1.

**Conclusion:**

Syndecan-1 immunoreactivity was decreased in the majority of human colon adenocarcinomas in correlation with TNM stage and metastasis to local lymph nodes. In a small fraction of adenocarcinomas, syndecan-1 was upregulated in the local stroma. Syndecan-1 expression status did not correlate with patient survival outcomes. Combined analysis of syndecan-1 in relation to a potential prognostic biomarker, fascin, identified that loss of tumor syndecan-1 correlated significantly with strong stromal fascin staining.

## Background

Syndecans are a conserved family of transmembrane heparan sulfate proteoglycan receptors that participate in cell-cell and cell-extracellular matrix adhesion and the actions of peptide growth factors in normal tissues [1,2]. In land vertebrates, the syndecan gene family contains 4 members of which syndecan-4 is the most widely expressed [3]. Syndecan-1 is expressed predominantly in epithelia, but is also found on fibroblasts, myoblasts and differentiating B cells and is up-regulated in multiple myeloma [1,2,4]. Syndecan-1 null mice are resistant to Wnt1-activated mammary tumors and carcinogen-induced tumor development and have increased susceptibility to allergen-induced airway inflammation [5-7]. Syndecan-1 null mice are also defective in repair of corneal and epidermal wounds [8,9]. Overall, these data demonstrate important contributions of syndecan-1 in the regulation of epithelial homeostasis, proliferation and migration.

In cell culture models, E-cadherin dependent loss of cell-surface syndecan-1 is associated with epithelial-mesenchymal transition and the epithelial phenotype is restored by over-expression of syndecan-1 [10-12]. Syndecan-1 over-expression also inhibits cell invasion into collagen gels [13]. Similarly, malignant transformation of Caco-2 epithelial cells is associated with loss of syndecan-1 [14]. Although these studies implicated tumor suppressor roles of syndecan-1, the relationship of syndecan-1 expression to tumor progression or clinical outcomes in human carcinomas has proved to be more complex. Thus, in gastric cancers, tumor cell expression of syndecan-1 has been correlated with patient survival and the loss of syndecan-1 with a poor prognosis [15,16]. A fraction of patients have abnormal up-regulation of syndecan-1 on stromal cells in the vicinity of the tumor, and this also correlates with poor prognosis [16]. Similar observations have been made in other carcinomas, for example [17-20]. However, for pancreatic and breast carcinomas there are conflicting reports that increased syndecan-1 expression correlates with a poor prognosis or resistance to chemotherapy [21-24]. These complex data point to a need for continuing assessment of syndecan-1 status in relation to the clinico-pathological characteristics of carcinomas from different tissue sources.

Colorectal carcinoma is the third most common form of cancer in both men and women in the USA and Europe. It remains a major cause of cancer mortality, with a 5 year survival rate of 60%, and its incidence is expected to increase in association with the ageing of western populations [25]. The major therapeutic approach is surgical resection and there is an urgent need to identify new biomarkers to improve strategies for adjuvant therapies or post-operative monitoring. We and others have recently demonstrated that expression of the actin-bundling protein, fascin, has prognostic significance in colorectal adenocarcinoma [26,27]. In non-transformed cells, syndecan-1 acts as a transducer of extracellular matrix cues that regulate the organization of actin and fascin in lamellipodia [28]. It has been reported that syndecan-1 expression is decreased in colorectal adenocarcinomas in comparison to adenomas and the normal tissue [29-31], and that reduced expression correlates with the incidence of local metastases [30]. Increased levels of syndecan-1 in the local stroma have also been described [32]. However, the prognostic relevance of changes in syndecan-1 expression in colorectal carcinoma remains unclear from the published studies [30,31]. In view of the health burden imposed by colorectal carcinoma, we examined a tissue microarray of clinically annotated colorectal carcinoma specimens and report on our analysis of epithelial or stromal syndecan-1 expression in relation to patient outcomes and the expression of the recently identified potential biomarker, fascin.

## Methods

### Immunohistochemical staining

The tissue microarray (TMA) was custom built and validated as described [26]. The TMA contained a total of 374 cores, from 14 normal colonic epithelia, 15 adenomas and 158 colorectal adenocarcinomas that were diagnosed at The Cleveland Clinic between 1993 and 1999. The majority of the adenocarcinomas were moderately differentiated and six were poorly-differentiated. Tumors were classified according to standard TNM staging guidelines [33]. To minimize sampling errors, two separate large diameter (1.5 mm diameter) tissue cores of each adenocarcinoma were included in the array, totaling a surface area of 3.5 mm^2 ^per case. The areas covered by these cores included the edge of each tumor. Each separate tissue core was assigned a unique TMA location number that was linked to a CCF Institutional Review Board-approved (IRB-5085) database containing a mean 38 months of clinical follow-up. From the 316 cores, 131 of the 158 adenocarcinoma samples were available for scoring. Immunohistochemistry was carried out using an automated Ventana Benchmark system as described [26]. Briefly, a 4-μm thick unstained section of each TMA was placed onto an electrostatically charged glass slide and baked for tissue adherence. Slides were pretreated with the recommended pretreatment solution (Ventana) for tissue deparaffinization and antigen retrieval. Slides were incubated with clone B-A38 mouse monoclonal antibody to human syndecan-1/CD138 (Serotec) at 1: 100 dilution, a secondary biotinylated antibody and a streptavidin amplification step. Antigen detection was carried out by peroxidase/3,3'-diaminobenzidine reaction. CD138 immunoreactivity was scored by two independent observers in a blinded procedure without prior knowledge of the clinical information and was classified for the staining of the tumor or the stromal cells, in either the tumor or the stroma, as 0 (less than 5% of cells); 1+ (5% – 25% of cells); 2+ (25% – 75% of cells), or 3+ (more than 75% of cells). Positive staining in plasma cells served as an internal positive control for syndecan-1. Stromal positivity was evaluated for the non-plasma cell stromal elements. In the less than 10% of cases where the opinions of the two evaluators differed, a consensus agreement was reached by re-review of the slides, thorough discussion and if necessary taking the average score. The fascin staining and scoring method has been described [26].

### Statistical analysis

The expression level of CD138 in relation to clinicopathological factors, Ki67 index, or fascin expression was analyzed using chi^2^. Overall survival was defined as that from the date of the operation to the date of death due to cancer. The Kaplan-Meier method was used to determine the probability of survival and data was analyzed with the log-rank test. StatView for Windows version 5 software (SAS Institute, Cary, NC) was used for the analysis. Median survival times were calculated using Dr. SPSS II for Windows version 11.0.1J (SPSS Japan Inc., Tokyo, JAPAN). In the analyses, a *p *value of <0.05 was considered significant.

## Results

### Syndecan-1 expression in colon adenocarcinomas

**Figure 1:**
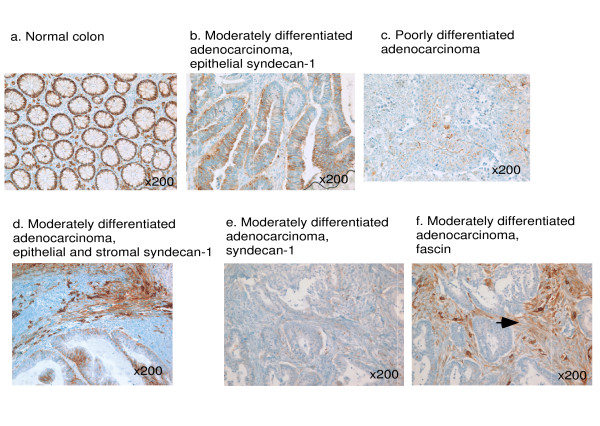
**Expression of syndecan-1 in normal colonic epithelium and colon adenocarcinomas**. a, immunohistochemical staining for syndecan-1 in normal colon. b-c, syndecan-1 staining of colon adenocarcinomas is lost in correlation with tumor differentiation status. d, example of a moderately differentiated adenocarcinoma with strong stromal staining for syndecan-1 and moderate staining of the tumor. e, f, contiguous sections of the same tumor stained for syndecan-1, (e), or fascin, (f), demonstrating strong staining for fascin in the stroma (arrowed). The tumor cells are negative for fascin and syndecan-1. Isolated strongly fascin positive cells correspond to dendritic and vascular endothelial cells and provideinternal positive controls for the fascin staining.

In the normal colon, syndecan-1 was expressed around the basolateral membrane of the normal columnar epithelium and in plasma cells. Syndecan-1 staining was absent from the stroma (Fig. 1a). This staining pattern was unchanged in adenomas (not shown). In contrast, in the majority of the adenocarcinomas, syndecan-1 staining was decreased or absent (Fig. 1b,c). Loss of syndecan-1 from tumor cells was most pronounced in the most poorly differentiated tumors (Fig. 1c). Out of 131 patients, in 65 cases syndecan-1 was expressed on less than 25% of the tumor cells, and in 49 cases between 25% to 75% of tumor cells were stained. Only in 17 cases did more than 75% of the tumor cells stain for syndecan-1 (Table 1). On the tumor cells, syndecan-1 was localized around the entire cell membrane and in many cells appeared to be cytoplasmically located (Fig. 1b). The TMA cores included the tumor borders, however we did not detect any consistent specific association of syndecan-1 immunoreactivity with tumor edges.

**Table 1:** Relationship between syndecan-1 immunoreactivity and clinicopathological characteristics

	Syndecan-1 staining (percentage of tumors in brackets)				
	0	1+	2+	3+	
	n = 38 (29%)	n = 27 (21%)	n = 49 (37%)	n = 17 (13%)	*p*
Variable					
Age					0.651
<65 yrs	16 (42.1)	12 (44.4)	17 (34.7)	6 (35.3)	
≥ 65 yrs	22 (57.9)	15 (55.6)	32 (65.3)	11 (64.7)	
Gender					0.042*
Male	24 (63.2)	22 (81.5)	23 (46.9)	10 (58.8)	
Female	14 (36.8)	5 (18.5)	26 (53.1)	7 (41.2)	
Stage					0.045*
I/II	14 (36.8)	13 (48.1)	33 (67.3)	9 (52.9)	
III/IV	24 (63.2)	14 (51.9)	16 (32.7)	8 (47.1)	
Lymph node metastasis					0.017*
negative	16 (42.1)	14 (51.9)	36 (73.5)	12 (70.6)	
positive	22 (57.9)	13 (48.1)	13 (26.5)	5 (29.4)	
Location					0.380
Proximal	13 (34.2)	9 (33.3)	13 (26.5)	2 (11.8)	
Distal	25 (65.8)	18 (66.7)	36 (73.5)	15 (88.2)	

In contrast to the normal stroma that was typically negative for syndecan-1 (Fig. 1a), some tumor specimens, without or with tumor expression of syndecan-1, had positive staining for syndecan-1 in the stroma. In total, 16.6% of the tumors had some level of stromal syndecan-1 staining: 8.8% of tumors had weak expression and 7.9% had moderate or strong expression. Fig. 1d shows an example of a tumor with moderate tumor syndecan-1 and strong stromal staining for syndecan-1.

### Relationship between syndecan-1 status, clinicopathological characteristics and clinical prognosis

**Figure 2:**
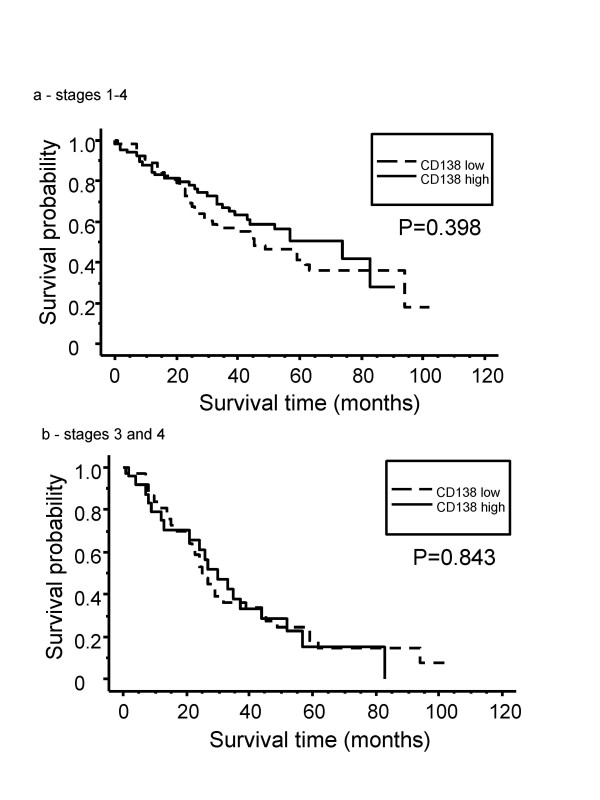
**Relationship of tumor syndecan-1 status to patient survival**. The Kaplan-Meier survival curves demonstrate that low (0 or +1 staining) or high (+2 or +3 staining) syndecan-1 staining of the tumors did not correlate with overall survival, either in the whole study population (a) or the stage III/IV tumors (b).

The correlation between syndecan-1 immunoreactivity and several clinico-pathological characteristics was investigated. The TMA had been previously validated as representative for traditional prognostic variables of colorectal cancer, including the correlation of tumor stage and metastasis to local lymph nodes with patient survival [26]. The incidence of low expression of syndecan-1 was significantly higher in males (p = 0.042), in tumors of higher TNM stage (p = 0.045), and in patients positive for lymph node metastasis (p = 0.017) (Table 1). There was no correlation between syndecan-1 status and tumor location (Table 1). In cumulative survival analysis, there was no difference of prognosis associated with either low or high syndecan-1 on the tumor cells or in the stroma. This result was obtained when each scoring category was examined individually, or when the samples were compared as two groups (low, corresponding to 0 and 1+ scores versus high, corresponding to 2+ and 3+ scores) using the Kaplan-Meier method. This result was obtained when the analysis for tumor syndecan-1 was applied either to the whole group of stage I-IV cases (p = 0.398), or to the stage III-IV group alone (p = 0.843) (Fig. 2; for clarity of presentation only the data for the low vs. high scoring groupings is shown). We also examined the 5 year survival rate and median survival times for each of the tumor syndecan-1 scoring categories. Again, there was no significant difference in survival between the scoring categories (Table 2).

**Table 2:** Relationship between tumor syndecan-1 status and patient 5 year and median survival

Syndecan-1 Scoring	5 yr survival rate (%)	Median survival (months)	95% CI
0	38.6	43	21–97
1+	47.7	49	16–82
2+	52.0	83	45–121
3+	43.7	57	5–109

### Relationship between syndecan-1 status and other biomarkers

We had previously examined the same tissue microarray set of tumors for fascin and Ki67 immunostaining. Positive fascin expression correlated with a poor prognosis in stage III/IV patients and a high Ki67 index correlated with TNM stage [26]. Because syndecan-1 and fascin are components of the same pathway that regulates cell motility in non-transformed cells, the syndecan-1 stained samples were evaluated for possible correlations with either fascin status or Ki67 index, with reference to our previously published datasets for these markers. There was no correlation between low expression of syndecan-1 and high fascin expression in tumor cells, either in the whole group or the stage III/IV patients alone. Neither tumor nor stromal syndecan-1 correlated with Ki67 index (data not shown). However, reduced tumor cell staining for syndecan-1 correlated significantly with strong stromal staining for fascin (*p *= 0.016; Table 3). An example of a syndecan-1 negative tumor with strong stromal staining for fascin is shown in Fig. 1e and 1f.

**Table 3:** Loss of tumor cell syndecan-1 correlates with high stromal fascin staining

	Fascin in stroma	
Tumor CD138	(0/2+)	(3+)
CD138 (0/1+)	55	10
CD138 (2+/3+)	64	2
p value	nsd	0.0164

## Discussion

The results of our study demonstrate that the loss of expression of syndecan-1 from colonic epithelial cells in colorectal adenocarcinomas correlates with tumor TNM stage and incidence of local lymph node metastasis but nevertheless does not correlate statistically with patient survival. Two previous studies reached conflicting conclusions on whether reduced syndecan-1 correlated with decreased patient survivial [30,31]. Our data are in agreement with the study of Lundin et al. [31]. We also examined the relationship between syndecan-1 and a recently identified novel independent prognostic factor for colorectal carcinoma, fascin [26,27]. A novel significant correlation between decreased syndecan-1 staining on tumor cells and increased stromal fascin staining was detected.

Our findings from a large, clinically annotated tissue microarray of colorectal carcinoma specimens add to the body of evidence that loss of epithelial syndecan-1 is a general feature of carcinoma progression. In agreement with other analyses of colorectal carcinoma, loss of epithelial syndecan-1 correlated with tumor TNM stage [29-31] and incidence of metastases to local lymph nodes [30,31]. Epithelial to mesenchymal transition (EMT) is a major process in tumor progression and loss of expression of syndecan-1 is well established to regulate aspects of EMT [1,10,11]. Thus the loss of epithelial syndecan-1 is likely permissive for development of higher stage, more biologically aggressive tumors. The correlation of loss of syndecan-1 with male gender documented in our study (Table 1) has not been observed in previous studies and the biological significance of this observation is unclear at this time. Loss of syndecan-1 did not correlate with tumor location. Thus there does not appear to be a close biological relationship between syndecan-1 status and microsatellite instability, which is strongly associated with tumor location in the proximal colon [34].

In a minority of the specimens (16.6%) stromal staining for syndecan-1 was increased in comparison to the normal tissue. This percentage is markedly lower than in one prior report, where stromal syndecan-1 immunoreactivity was observed in 58% of specimens [31], but is consistent with an analysis of micro-dissected tissues that identified 7.4% of specimens to have elevated syndecan-1 content, largely due to stromal expression [32]. By comparing *in situ *hybridization with immunohistochemistry, the latter study demonstrated that stromal syndecan-1 immunoreactivity is due to expression of the SDCN1 transcript in a stromal cell population, likely myofibroblasts [32]. It is likely that stromal syndecan-1 has impact on the tumor microenvironment by alterations to the retention of heparin binding growth factors and extracellular matrix components in the vicinity of the tumor. These factors have been proposed to facilitate tumor cell invasion and, in general, the tumor stroma has an important role in cancer development [1,4,35]. Nevertheless, from the current analysis, the presence of stromal syndecan-1 did not correlate with a more biologically aggressive tumor phenotype or altered patient survival outcomes. A previous analysis is consistent with this conclusion [31]. We observed a wide variation of staining intensity for stromal syndecan-1 and also variations in the distribution of staining, which tended to be patchy within limited areas of the stroma. We speculate that expression of stromal syndecan-1 might be transient and reflective of short-term phenotypic changes in stromal myofibroblasts.

Although our data did not support syndecan-1 status as an independent prognostic factor in colorectal carcinoma, it was of interest to examine whether additional information could be obtained from analyzing syndecan-1 status in combination with another candidate biomarker. We selected fascin for this analysis, because syndecan-1 is a functionally significant regulator of the cytoskeletal organization of actin and fascin in several normal cell types [28,36]. Moreover, fascin is absent from normal colonic epithelium and its upregulation in colorectal adenocarcinomas correlates with poor prognosis [26,27]. In the normal stroma, fascin is detected at low levels in fibroblasts and at higher levels in dendritic cells and vascular endothelial cells. These studies also uncovered that stromal fascin is increased in at least 47% of tumor specimens, irrespective of the fascin status of the tumor [26,37]. From study of contiguous sections of the same Cleveland Clinic colorectal tissue microarray specimen set stained for syndecan-1, we identified that loss of tumor cell syndecan-1 did not correlate with the upregulation of fascin in clinically aggressive adenocarcinomas. However, loss of syndecan-1 immunoreactivity of the tumor did correlate significantly with strong stromal fascin staining. This novel finding brings further support to the idea that upregulation of stromal fascin may represent an aspect of the host-tumor interaction. The altered adhesive and motility properties of both tumor cells and adjacent stromal cells may jointly contribute to tumor progression. Future studies of independent datasets will be needed to validate the statistical as well as the clinical significance of the combined biomarker data.

We also identified that, in the tumors where stromal syndecan-1 was elevated, stromal syndecan-1 frequently overlapped with areas of increased stromal fascin (Fig. 1d). However, the fractions of positive specimens (16.6% for stromal syndecan-1 and 47% for stromal fascin) were very different. We infer that either the two molecules are under separate regulation, or that fascin is expressed by multiple cell types within the stroma. Abnormal expression of fascin by foci of stromal fibroblasts has also been observed in idiopathic pulmonary fibrosis [38]. We speculate that for stromal cells in which both syndecan-1 and fascin are upregulated, syndecan-1 could provide pro-migratory cues through its intracellular regulation of fascin that promotes formation of lamellipodia [28]. On the basis of the small total number of cases that were positive for stromal syndecan-1 in our dataset, the observation of co-staining for stromal syndecan-1 and fascin did not have statistical significance.

## Conclusion

Syndecan-1 immunoreactivity is decreased in the majority of human colon adenocarcinomas in correlation with TNM stage and local lymph node metastasis. A small fraction of adenocarcinomas have increased syndecan-1 staining in the local stroma. Syndecan-1 status does not correlate with patient survival outcomes. Combined analysis of syndecan-1 in relation to a recently identified potential prognostic biomarker, fascin, identified a subset of tumors in which loss of tumor cell syndecan-1 correlates significantly with up-regulation stromal of fascin. These findings may assist improved biomarker identification of aggressive forms of colorectal adenocarcinoma.

## Competing interests

The authors declare that they have no competing interests.

## Authors' contributions

MS organized the stainings, scored sections and contributed to the study design and drafting of the manuscript. YH scored sections, carried out the statistical analysis, prepared figure panels and the Table and contributed to the drafting of the manuscript. JCA designed the study, participated in data analysis, prepared Fig. 1 and drafted the manuscript. Allauthors read and approved the final manuscript.

## Pre-publication history

The pre-publication history for this paper can be accessed here:


